# Addressing the Digital Divide Among the Older Population Presents a Substantial Challenge

**DOI:** 10.2196/69482

**Published:** 2025-03-28

**Authors:** Su-hang Xie

**Affiliations:** 1 Department of Rehabilitation Medicine The First Medical Center of PLA General Hospital Beijing China

**Keywords:** exergame training, Matter of Balance, MOB, pre-frail, tele-exergame, tele-rehabilitation, gaming-based, tele-exercise, physical function, frailty, older adults, aging, physical activity, dementia, CogXergaming, telehealth, dynamic balance

We are pleased to acknowledge the recent publication of the study by Kannan et al [1] in the *Journal of Medical Internet Research*. The study’s findings offer valuable insights into the potential for more tailored teletherapy or support interventions for older adults, particularly in the context of rapid technological advancements in health care. Of the 50 older adults initially recruited, 31 successfully completed the full program, yielding a completion rate of approximately 60%. One of the primary reasons for withdrawal was a lack of interest in the content of the intervention. This observation raises the concern that, despite the pervasive presence of advanced technologies in modern life, certain older individuals may be effectively left behind. This phenomenon, known as the digital divide, has garnered increasing attention in recent years [2]. The digital divide refers to the disparity between individuals who have access to and the skills to use information and communication technology (ICT) and those who do not [3]. Although first recognized in the previous century, the digital divide is becoming more pronounced in the context of rapid technological advancements, particularly the frequent updates to ICT. The digital divide has a particularly profound impact on older populations, with many rapidly developing countries experiencing an increase in both the absolute and relative number of individuals affected by the digital divide [4].

In light of the rapid advances in modern medicine, how might we effectively address the digital divide in the older population? It is our contention that the older population should be permitted to transition from a position of passive recipient to that of active initiator and designer of projects. This would enable them to move from being the object of attention to becoming active problem-solvers and contributors to the development process. Before developing a teleprogram application, older adults should be engaged as co-designers, providing feedback from a problem-solving perspective. This ensures that projects are developed from their viewpoint and that they are not treated as passive recipients in the final clinical trial. It is therefore recommended that in future work building on the study by Kannan et al [1], frail older individuals be included as coresearchers in the development and refinement of the CogXergaming program. This inclusion would not only enrich the project but also enhance its generalizability and relevance to the frail older population. Furthermore, Kannan et al [1] did not perform a correlation analysis between the participants’ years of education, as reported in the participant demographic characteristics, and their ability to learn effectively—an important factor in addressing the digital divide among older adults. Future studies exploring the correlation between education, learning curves, and application outcomes could provide valuable insights into overcoming the digital divide. In conclusion, CogXergaming presents a novel approach to intervention for frail, older individuals, and it will be of great interest to explore how this intervention can contribute to bridging the digital divide among older adults.

## Abbreviations

ICT: information and communication technology
